# Improving deep‐learning IMRT plan quality for lymphoma using deliverability‐aware training

**DOI:** 10.1002/acm2.70815

**Published:** 2026-10-05

**Authors:** Denis Kutnár, Ivan Richter Vogelius, Hristo Atanasov Georgiev, Lena Specht, Jens Petersen

**Affiliations:** ^1^ Department of Computer Science University of Copenhagen Copenhagen Ø Denmark; ^2^ Department of Oncology Rigshospitalet Copenhagen Ø Denmark; ^3^ Faculty of Health and Medical Sciences University of Copenhagen Copenhagen N Denmark

**Keywords:** Automated treatment planning, cycle consistency, deep learning, fluence prediction, IMRT, plan deliverability

## Abstract

**Background:**

Deep learning (DL) methods have shown strong performance in predicting dose distributions from patient anatomy and target structures; however, accurate dose prediction does not guarantee a deliverable plan after conversion into machine parameters and dose recalculation in the treatment planning system (TPS), motivating training objectives that explicitly couple prediction to deliverability. Lymphoma presents substantial clinical heterogeneity in target location, extent, and prescription/fractionation, so fast automated methods that produce deliverable plans are particularly valuable.

**Purpose:**

To investigate whether a deliverability‐aware training framework improves the end‐to‐end deliverability of DL‐generated intensity‐modulated radiotherapy (IMRT) plans for lymphoma.

**Methods:**

A retrospective single‐institution dataset of 619 plans (571 patients; 2009–2021) was used. Clinical volumetric modulated arc therapy (VMAT) plans were converted to standardized 15‐field coplanar sliding‐window IMRT reference plans in Eclipse using automated scripting. Two DL approaches were compared: a baseline dose‐only objective model (DL‐DO) and a multi‐objective model (DL‐MO) trained with dose supervision, fluence supervision, and a dose–fluence–dose cycle‐consistency constraint implemented via fixed inverse and forward networks. Predicted fluence maps were imported into the TPS, leaf‐sequenced, and recalculated with AcurosXB without further optimization; plan quality was evaluated on a held‐out test set (*n* = 61) using planning target volume (PTV) *D*_98_, *D*_2_, and *D*_max_ with paired Wilcoxon testing and Holm correction.

**Results:**

DL‐MO produced significantly better machine‐deliverable plans than DL‐DO, improving coverage and reducing hotspots (median paired differences: +12.31 percentage points (pp) in *D*_98_, −3.43 pp in *D*_2_, and −3.18 pp in *D*_max_; Holm‐corrected *p* < 0.0001 for all). Mean ± SD PTV metrics were *D*_98_ = 90.10 ± 3.74% vs. 78.59 ± 5.92%, *D*_2_ = 106.28 ± 1.13% vs. 109.65 ± 2.40%, and *D*_max_ = 110.32 ± 2.20% vs. 113.27 ± 3.03% (DL‐MO vs. DL‐DO). DL‐MO approached the TPS reference IMRT plans (*D*_98_ = 91.62 ± 3.45%) with residual hotspot elevation.

**Conclusions:**

Cycle‐consistency training improves the end‐to‐end deliverability of DL‐generated IMRT plans for heterogeneous lymphoma, yielding substantially better TPS‐recalculated, machine‐deliverable plans than conventional dose‐only supervision while maintaining sub‐second fluence‐map generation. However, statistically significant differences from the TPS reference remained for *D*_98_, *D*_2_, and *D*_max_.

## 1 INTRODUCTION

Intensity‐modulated radiation therapy (IMRT) planning remains labor‐intensive and difficult to standardize, with plan quality often depending on iterative adjustment of optimization objectives and the experience of the planner.^1^, ^2^ This motivates approaches that improve planning consistency and efficiency.^1^, ^3^ These challenges are amplified in lymphoma, where target extent can be large and anatomically diverse and prescription strategies vary across the clinical spectrum, increasing planning burden and inter‐planner variability.^4^, ^5^

Deep learning (DL) methods have shown strong performance for predicting dose distributions directly from patient anatomy and target structures.^6^, ^7^, ^8^, ^9^ Most work in this area focuses on supervized learning of a reference dose distribution, typically by minimizing voxel‐wise errors between predicted and planned dose.^7^, ^8^, ^9^, ^10^ While such models can generate plausible dose patterns, accurate dose prediction alone does not guarantee that the prediction is deliverable after conversion into machine parameters and dose recomputation in the treatment planning system (TPS).^11^

Several groups have evaluated end‐to‐end pipelines in which predicted dose distributions or fluence maps are used as intermediate inputs to downstream plan generation, including dose mimicking or inverse planning.^10^, ^12^, ^13^, ^14^, ^15^, ^16^, ^17^ Because deliverable modulation is determined by subsequent optimization and sequencing, the recalculated dose after TPS processing can deviate from the raw prediction and exhibit degraded dosimetric quality, even when prediction metrics appear favourable.^10^, ^12^, ^13^, ^16^ This motivates learning objectives that tie the prediction explicitly to the steps required to obtain a deliverable plan, rather than optimizing only a direct similarity to a reference dose.^14^, ^15^, ^16^, ^18^, ^19^

Cycle‐consistency constraints have been used in DL for radiotherapy (RT), particularly for cross‐modality image translation tasks such as synthetic computed tomography (CT) generation and cone‐beam CT correction.^20^, ^21^ However, the use of cycle consistency as an explicit constraint in RT planning has not been previously reported.

We investigate whether incorporating cycle consistency can improve the deliverability of DL‐generated IMRT plans for lymphoma. To test this, we compare TPS reference plans with DL plans generated by two distinct models: a baseline dose‐only objective model (DL‐DO) and a multi‐objective model (DL‐MO) that includes a dose–fluence–dose cycle‐consistency term.

## 2 METHODS

### 2.1 Methods overview and study design

We compared TPS reference IMRT plans with plans generated using two DL models: a baseline DL‐DO and a DL‐MO trained with dose supervision, fluence supervision, and cycle consistency. Figure 1 summarizes the proposed framework. The primary endpoint was the final machine‐deliverable TPS plan obtained by importing the predicted fluence maps for leaf sequencing followed by AcurosXB dose recalculation for both DL methods.

**FIGURE 1:**
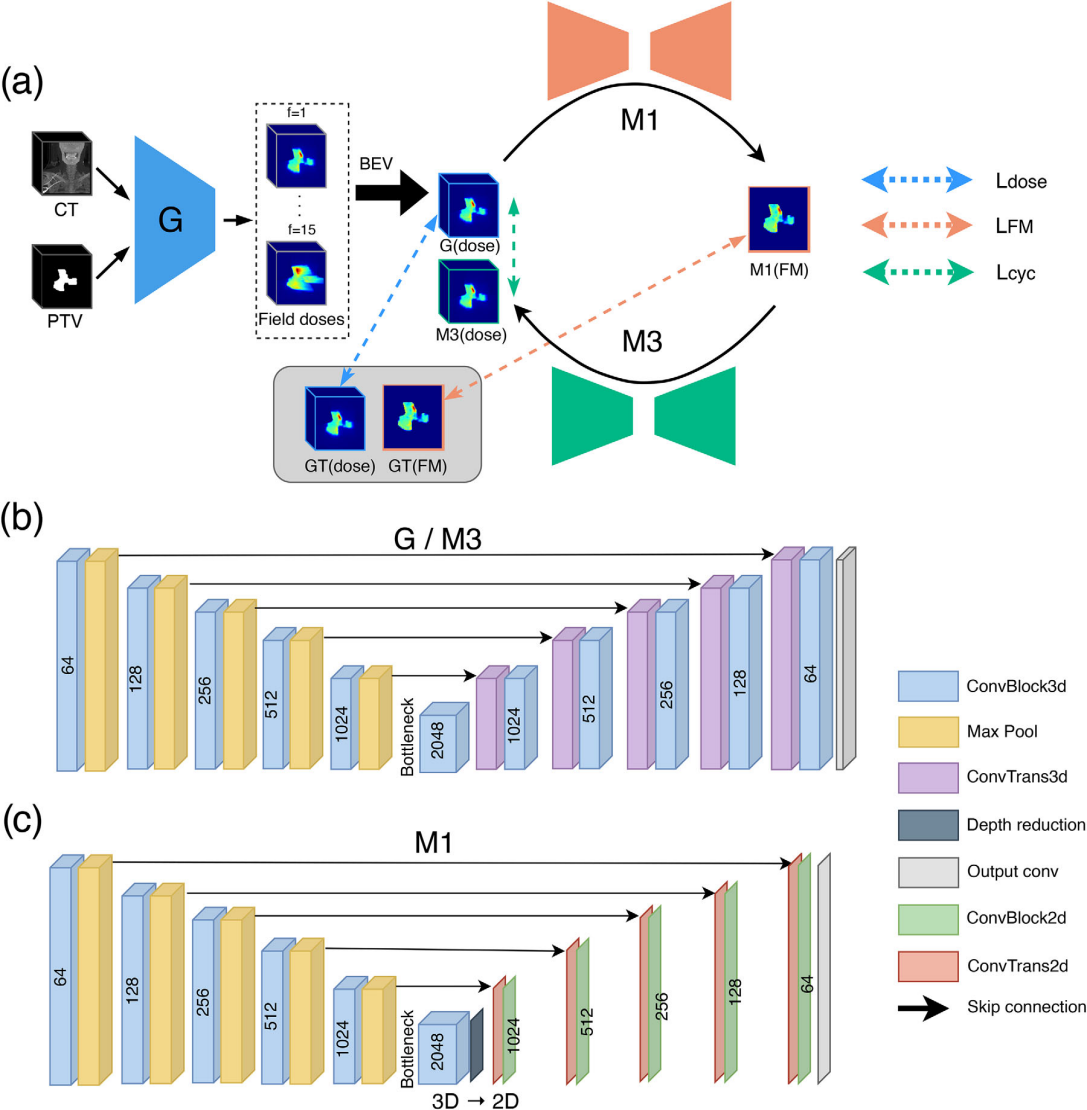
Overview of the deliverability‐aware training framework and network architectures. (a) Workflow overview showing the dose–fluence–dose loop and the three training losses, L_dose_, L_FM_, and L_cyc_, which are used to update the generator G. (b) Shared 3D encoder–decoder architecture used by G and M_3_. (c) 3D encoder–2D decoder architecture of M_1_. Numbers indicate the number of feature channels. CT, computed tomography; PTV, planning target volume; BEV, beam's‐eye view; FM, fluence map; GT, ground truth.

### 2.2 Dataset and reference plan generation

We retrospectively assembled a lymphoma RT dataset from a single institution under ethical approval (R‐23054402). The cohort comprised 619 treatment plans from 571 patients treated between 2009 and 2021, with most patients contributing a single plan (median 1, range 1–3). Of these plans, 522 were prescribed with curative intent and 97 were palliative. The population included 334 males and 237 females, with a median age of 73 years (range 26–103). Target size varied substantially across cases, reflecting the anatomical diversity typical of lymphoma: the PTV volume had a median of 195 cm^3^ (range 5.4–2,282 cm^3^). The anatomical treatment sites were distributed across the head and neck (222 plans, 35.9%), thorax (181 plans, 29.2%), abdomen (121 plans, 19.5%), multiple regions (38 plans, 6.1%), pelvis (31 plans, 5.0%), intracranial/central nervous system (21 plans, 3.4%), and extremities (5 plans, 0.8%).

All patients were treated clinically with volumetric modulated arc therapy (VMAT) in Eclipse (Varian Medical Systems), using one or two arcs according to routine practice, with dose calculated using AcurosXB (326/619 plans) or the Anisotropic Analytical Algorithm (AAA, 293/619 plans). To obtain a consistent beam geometry for beam‐wise learning and standardized end‐to‐end evaluation, each clinical VMAT plan was converted to a reference IMRT plan using an automated Eclipse Scripting API (ESAPI)^22^, ^23^, ^24^ workflow in a research instance of Eclipse. The workflow created a 15‐field equi‐angular coplanar 6 MV sliding‐window IMRT plan for every case and transferred the clinical optimization objectives (including priorities and weights) from the VMAT plan to the IMRT optimization. The plan was then optimized, sequenced, and recalculated in the TPS. The 15‐field arrangement was selected to provide uniform angular sampling across heterogeneous lymphoma presentations and to enable beam‐wise modeling under a fixed geometry.

### 2.3 Data pre‐processing

All DICOM modalities (CTs, structures, field doses, and fluences) were exported from the TPS on an isotropic 2.5 mm grid, matching the resolution of the fluence maps. Volumes were rigidly aligned to the treatment isocentre to provide a common geometric reference across channels. Each modality was then cropped or padded to a fixed tensor size of 128 × 256 × 256 to enable batch training. For each beam, CT and field dose were transformed into beam's eye view (BEV) using a cone‐beam projection with a 100 cm source‐to‐axis distance. This representation accounts for beam divergence and makes the relationship between fluence modulation and deposited field dose more direct, aligning naturally with ray‐based physical interpretations of dose deposition. Field doses were stored in relative units, divided by the target prescription dose. CTs, field doses, and fluence maps were standardized using z‐scores with statistics computed on the training set. Patient‐level splits were applied prior to model development (train/validation/test: 495/63/61 plans), and the same pre‐processing was applied across all splits.

### 2.4 Model framework

The proposed framework comprises three neural networks that form a dose–fluence–dose loop. A generator *G* predicts the full set of beam‐wise field doses from patient anatomy, an inverse model *M*_1_ maps a single predicted field dose to a corresponding fluence map, and a forward model *M*_3_ maps that fluence map back to field dose.

To decompose the learning problem, we adopted a two‐stage approach, motivated by previous work.^14^, ^19^, ^25^ Predicting field doses as an intermediate representation enables direct dose‐domain supervision and allows the planning and physics components to be learned independently. The generator learns how anatomy and target structures relate to beam‐wise field doses, while the dose‐to‐fluence model learns the physical relationship between field dose and fluence. This separation is useful because clinical planning protocols may change over time, whereas the underlying physics is comparatively stable. The design also supports voxel‐wise or structure‐specific dosimetric losses and provides an interpretable intermediate output that can be inspected for anatomically and physically plausible dose‐deposition patterns. Finally, the field‐dose representation provides the basis for the dose–fluence–dose consistency constraint.

The models *M*_1_ and *M*_3_ operate in two modes. In the first mode, they are trained and evaluated on field doses and corresponding fluence maps optimized and derived from the TPS. In the second mode, they are used for cycle‐consistency training, where the input is the *G* prediction rather than the TPS‐optimized input. During this phase, the models remain frozen, while gradients are propagated through them to compute the training losses for *G*.

#### 2.4.1 Generator *G*

The generator *G* takes the planning CT and binary PTV mask as input and predicts all 15 IMRT field dose volumes in a single forward pass. The output is a stack of 3D field doses with one channel per beam. Predicting all fields jointly enables information sharing across beams and allows the network to learn trade‐offs between fields, reflecting the fact that modulation in one direction can compensate for limitations in another. The full dose can be obtained by summation of the 15 predicted field doses when required for evaluation. The network follows a 3D encoder–decoder architecture with skip connections; the encoder bottleneck aggregates global context across the full field of view, while skip connections preserve fine spatial detail needed for dose shaping.

The encoder comprised five downsampling stages with 64, 128, 256, 512, and 1024 feature channels, followed by a 2048‐channel bottleneck. Each encoder and decoder block contained two 3 × 3 × 3 convolutions with GroupNorm and ReLU activation. Downsampling was performed using 2 × 2 × 2 max pooling, while the decoder used five 2 × 2 × 2 transposed convolutions with stride 2 and skip‐feature concatenation. A final 1 × 1 × 1 convolution produced the 15 field‐dose channels. The network contained approximately 361.9 million parameters.

#### 2.4.2 Dose‐to‐fluence model *M_1_*

*M_1_* maps field dose to fluence. For each beam, it takes the planning CT and field dose, both in BEV, and predicts a 2D fluence map. It is trained to approximate the dose to fluence relationship realized by the TPS planning process within the fixed IMRT geometry, providing a differentiable estimate of the fluence implied by a predicted field dose.

*M_1_* used a five‐stage 3D encoder with 64, 128, 256, 512, and 1024 feature channels and a 2048‐channel bottleneck. Each encoder block contained two 3 × 3 × 3 convolutions with ReLU activation, with 2 × 2 × 2 max pooling between stages. The beam‐depth dimension was reduced at the bottleneck using a 1 × 1 × 8 convolution with stride 1 × 1 × 8, while the corresponding skip features were reduced using 1 × 1 × *k* convolutions, with *k* = 16, 32, 64, 128, and 256. The resulting features were processed by a five‐stage 2D decoder using 2 × 2 transposed convolutions with stride 2, skip concatenations, and blocks of two 3 × 3 convolutions. A final 1 × 1 convolution produced the fluence map. The network contained approximately 341.3 million parameters.

#### 2.4.3 Fluence‐to‐dose model *M_3_*

*M_3_* maps fluence to field dose. For each beam, it takes the BEV CT together with a fluence map and predicts a 3D BEV field dose. The fluence map is repeated along the beam axis to form a 3D volume with the same spatial dimensions as the field dose. Conceptually, *M_3_* serves as a learned analogue of photon dose calculation, approximating the mapping from fluence to deposited dose through anatomy in the BEV coordinate system.^26^, ^27^

*M_3_* used the same five‐stage 3D encoder–decoder architecture and convolutional configuration as *G*, but with a single output channel representing the reconstructed field dose. The network contained approximately 361.9 million parameters.

### 2.5 Training objectives and optimization

We trained two distinct DL models: a baseline dose‐only model (DL‐DO) using the loss L_dose_, and the multi‐objective model (DL‐MO) combining the loss L_dose_ with fluence supervision loss L_FM_ and a cycle‐consistency constraint enforced through the loss L_cyc_.

#### 2.5.1 Loss functions

*Dose loss*: voxel‐wise mean squared error (MSE) between the field doses predicted by the generator and the TPS reference field doses over all beams,


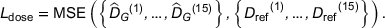




Here, 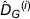 and ${D_{ref}}^{\left(i\right)}$ denote the generator‐predicted and TPS reference field dose for beam i ∈ {1, …, 15}.

*Fluence map loss*: pixel‐wise MSE between the TPS reference fluence map and the fluence predicted by the frozen M_1_, where M_1_ takes as input the generator‐predicted field dose for beam b together with the corresponding BEV CT,


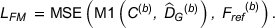




Where, $C^{\left(b\right)}$ denotes the corresponding BEV CT for beam b, 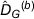 denotes the generator‐predicted field dose for beam b, and ${F_{ref}}^{\left(b\right)}$ denotes the TPS reference fluence map for beam b.

*Cycle‐consistency loss*: voxel‐wise MSE between the field dose predicted by the generator and the field dose reconstructed by M_3_ for beam b. The reconstructed dose is obtained by first mapping the predicted field dose into a fluence map using M_1_, conditioned on the corresponding BEV CT, and then mapping this fluence map back to field dose using the frozen M_3_,


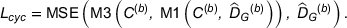




Here, 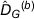 denotes the field dose predicted by the generator for beam b.

#### 2.5.2 Optimization strategy and training procedure

Multi‐objective training (DL‐MO) was initialized from the converged DL‐DO generator weights. To accommodate the change in objective, the optimizer state was reset and a dedicated learning rate was used. During DL‐MO optimization, the *M*_1_ and *M*_3_ models were held fixed: their parameters were not updated, but gradients were propagated through both networks. Early in training, predictions are generally poor and L_dose_ and L_FM_ are high and difficult to reduce. The cycle‐consistency, on the other hand, is trivially easy to satisfy for some dose distributions, such as zero doses. To avoid convergence to such trivial solutions and stabilize optimization, the cycle‐consistency loss was linearly ramped from 0.02 to 2.

Training plans were randomly shuffled each epoch to form mini‐batches. The dose loss L_dose_ was computed over all 15 predicted field doses for every plan. To reduce memory and computation, and to encourage beam‐wise generalization, the fluence mapping and cycle‐consistency terms were evaluated for a single beam selected at random per plan and iteration.

Learning rates between 10^−^^7^ and 10^−^^3^ were explored using a learning‐rate range test,^28^ and the final values were selected based on stable loss reduction and subsequent validation‐set performance. The batch size was set to the largest value permitted by GPU memory, resulting in a batch size of 8 with a learning rate of 10^−^^4^ for DL‐DO and a batch size of 3 with a learning rate of 10^−^^5^ for DL‐MO. For both models, early stopping and model selection were based on validation loss (L_dose_), with training stopped after 30 consecutive epochs without improvement. The final DL‐DO and DL‐MO models were trained for 980 and 370 epochs, respectively. Training was distributed across two GPUs, each with 48 GB of memory, with *G* placed on one GPU and *M*_1_ and *M*_3_ on the second.

#### 2.5.3 Ablation study

To isolate the contributions of fluence supervision and the dose–fluence–dose consistency loss, two additional training‐objective variants were evaluated. The first used L_dose_ + L_FM_, with the cycle‐consistency loss weight set to zero, while the second used L_dose_ + L_cyc_, with the fluence supervision loss weight set to zero. Both variants were initialized from the converged DL‐DO generator and followed the DL‐MO optimization procedure, including the same batch size, learning rate, frozen *M*_1_ and *M*_3_ networks, early‐stopping criterion, model‐selection procedure, and loss‐weight schedule for the retained loss component.

### 2.6 TPS‐based plan generation

Predicted fluence maps from both DL methods were converted to machine‐deliverable IMRT plans in Eclipse (Varian Medical Systems) using an automated ESAPI workflow in a research instance of the TPS. Plans were generated for delivery on a Varian TrueBeam linac (HD‐120 MLC, 6 MV). For each test case, the TPS reference IMRT plan was used as a template so that beam geometry, isocentre placement, and the structure set were identical across methods. The predicted fluence map for each field was imported into Eclipse, registered to the planning isocentre, transformed into the beam coordinate system, and assigned to the corresponding beam. Plans were then leaf‐sequenced using the clinical sliding‐window sequencer (Varian Leaf Motion Calculator v18.0.0), and dose was calculated using AcurosXB (v18.0.0) on the same dose grid. No further inverse planning, fluence re‐optimization, or manual editing was performed after import.

### 2.7 Evaluation and statistical analysis

All evaluations were performed on a held‐out test set using paired, per‐case comparisons. We compared TPS reference IMRT plans with plans generated using the two DL models, DL‐DO and DL‐MO, as illustrated in Figure 2a–c. The primary endpoint was the final machine‐deliverable TPS plan obtained from the predicted fluence maps of both DL methods. Plan quality was quantified on the PTV using dose–volume histogram (DVH) metrics for target coverage and hotspot control (*D_98_*, *D_2_*, *D_max_*), and the time required for fluence‐map generation was recorded. For comparability, all doses were renormalized to Dmean(*target*) = 100%.

**FIGURE 2:**
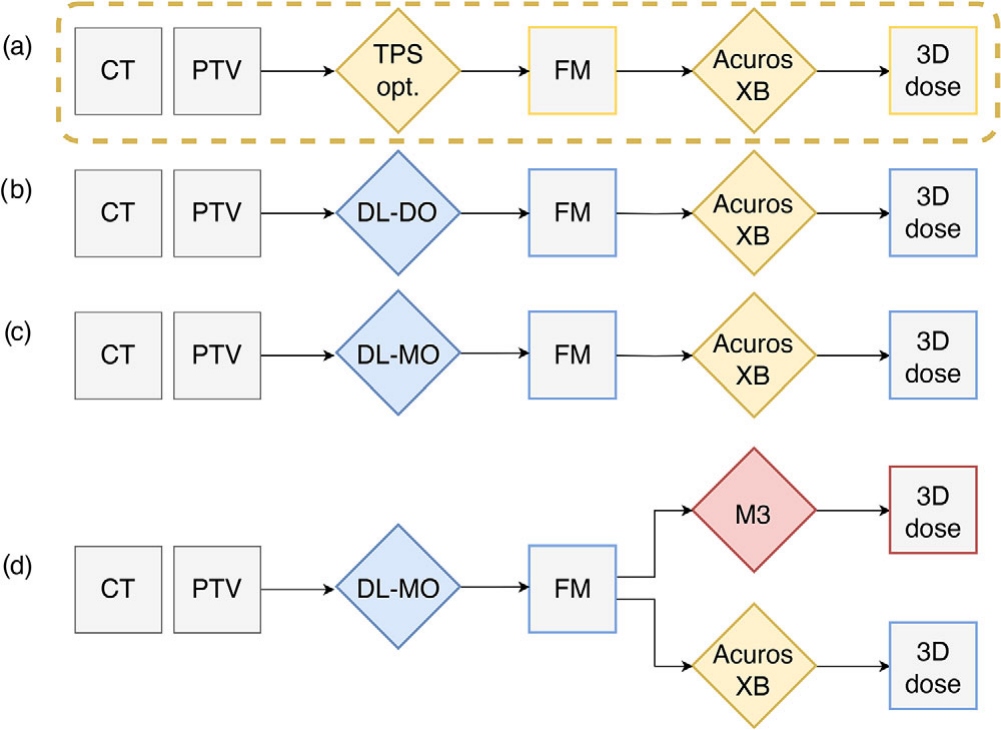
Overview of the evaluation framework. (a) Routine clinical TPS‐based planning workflow, used as the reference for final plan comparison and for stand‐alone evaluation of models M_1_ and M_3_. (b) Baseline DL‐DO and (c) proposed DL‐MO, both evaluated against the TPS reference plans in (a). (d) Separate evaluation of M_3_ on DL‐predicted FMs, in contrast to the TPS‐derived FMs in (a). CT, computed tomography; PTV, planning target volume; TPS, treatment planning system; DL, deep learning; FM, fluence map.

Exploratory dosimetric evaluation was performed for the most frequently delineated organs at risk (OARs), using *D_2_*% for the spinal cord and brainstem and Dmean for the parotid and submandibular glands. Metrics were extracted from the final TPS‐recalculated plans for all eligible cases and are reported descriptively as absolute dose in Gy, with *n* varying by OAR.

Models *M_1_* and *M_3_* were evaluated independently on paired field doses and corresponding fluence maps optimized and derived from the TPS (Figure 2a). Performance was assessed using pixel‐wise MAE/MSE for fluence maps (M_1_ output) and voxel‐wise MAE/MSE for field doses (M_3_ output). In addition, the accuracy of *M_3_* was evaluated on fluence maps generated by *G*, compared with the clinical dose calculation engine AcurosXB, to assess its performance under the conditions encountered during cycle‐consistency training (Figure 2d). Paired statistical testing used the Wilcoxon signed‐rank test with Holm correction for multiple endpoints; two‐sided *p* < 0.05 was considered statistically significant.

## 3 RESULTS

### 3.1 Stand‐alone evaluation of models *M*_1_ and *M*_3_

Because the proposed framework relies on *M*_1_ and *M*_3_ to impose cycle consistency during training, we first evaluated these models in a stand‐alone setting (Figure 2a). The *M*_1_ model (dose‐to‐fluence) reproduced TPS reference fluence maps with low pixel‐wise error (MAE = 0.015 ± 0.009, MSE = 0.013 ± 0.013) and good spatial agreement (Figure 3). The *M*_3_ model (fluence‐to‐dose) achieved low voxel‐wise error when predicting field dose from TPS fluence maps on the same held‐out test set (MAE = 0.029 ± 0.011, MSE = 0.012 ± 0.010) (Figure 4).

**FIGURE 3:**
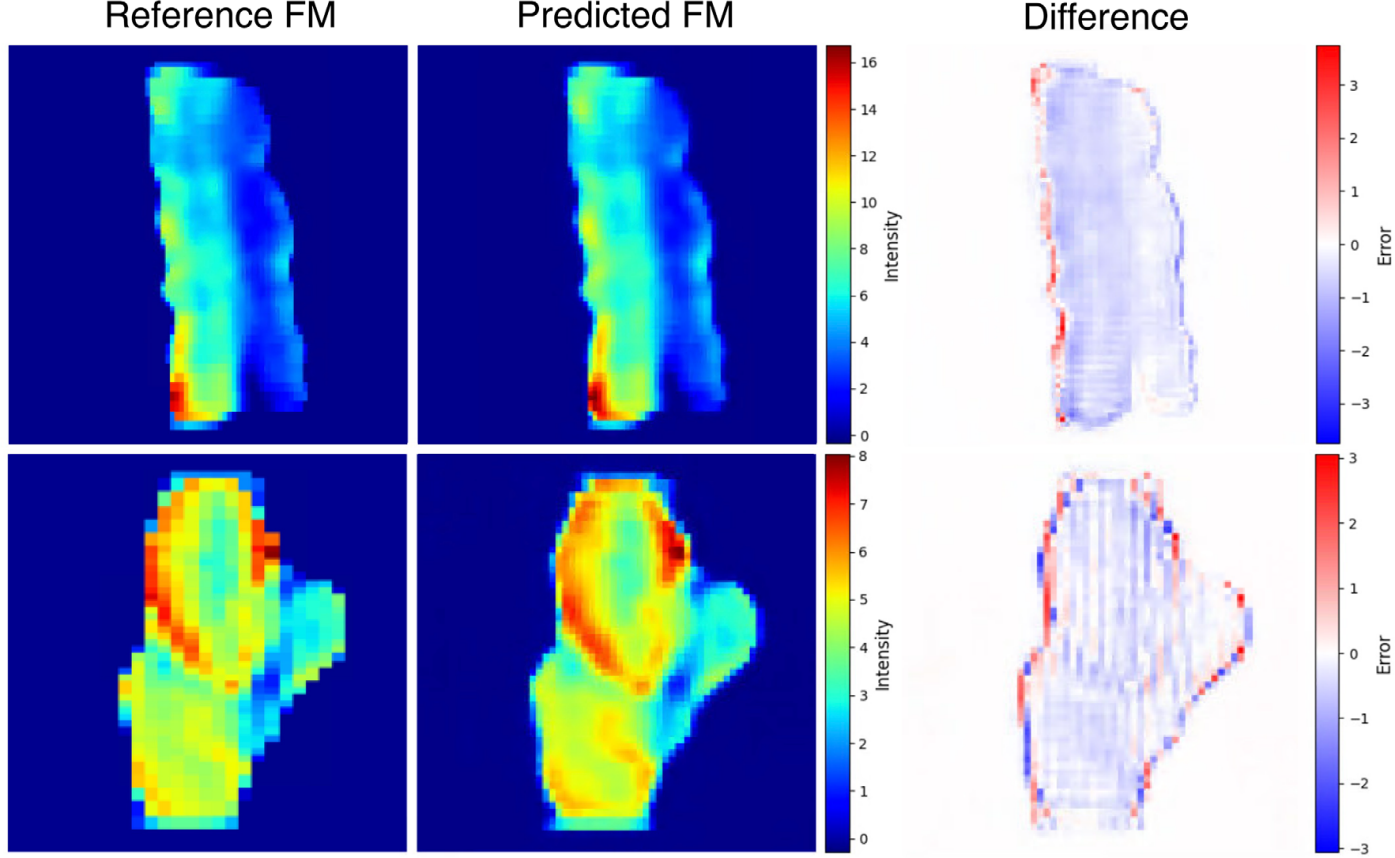
Stand‐alone evaluation of model M_1_ (dose‐to‐fluence), when trained on field doses and corresponding fluence maps optimized and derived from the TPS. Columns show the TPS reference fluence map, the M_1_‐predicted fluence map from the corresponding paired field dose, and the difference in relative units; rows show randomly selected test beams. TPS, treatment planning system.

**FIGURE 4:**
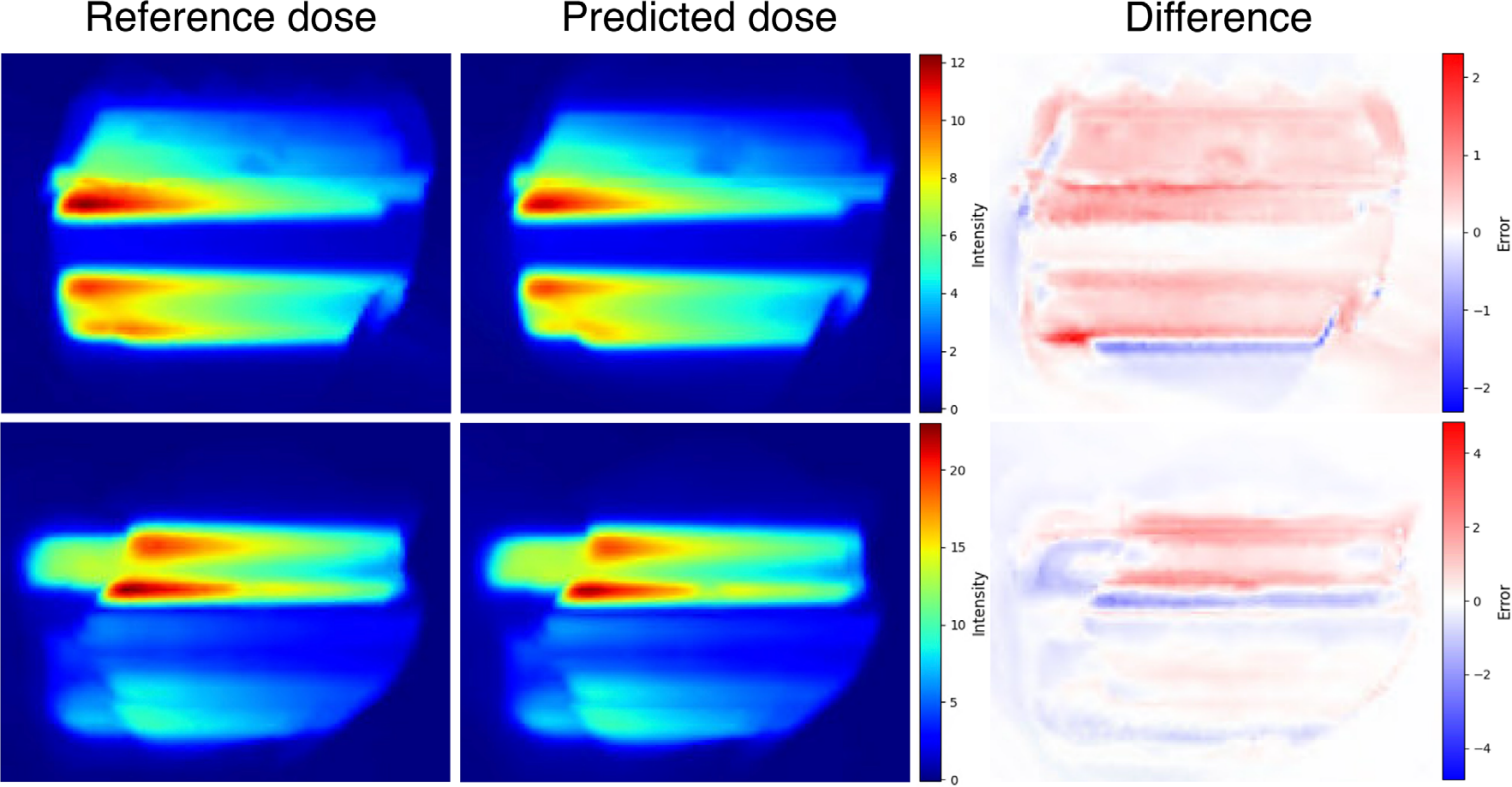
Stand‐alone evaluation of model M_3_ (fluence‐to‐dose), when trained on fluence maps and corresponding field doses optimized and derived from the TPS. Columns show the TPS reference field dose, the M_3_‐predicted field dose, and the difference; rows show randomly selected test beams displayed as MIPs relative to 100% of the target prescription. TPS, treatment planning system; MIP, maximum‐intensity projection.

### 3.2 End‐to‐end plan quality

For the end‐to‐end evaluation, we compared the final machine‐deliverable plans generated in the TPS for both DL methods (Figure 2b,c). These plans were obtained by importing the predicted fluence maps for leaf sequencing and dose calculation. The fluence maps predicted by DL‐MO more closely matched the TPS reference than those predicted by DL‐DO (Figure 5). For the three randomly selected beams displayed in Figure 5, the mean fluence‐map MSE was 8.36×10^−^^5^ for DL‐DO and 4.05×10^−^^5^ for DL‐MO, compared with 9.62×10^−^^5^ and 7.01×10^−^^5^, respectively, across the full fluence‐map evaluation set of 915 beam maps. The top and middle examples had lower‐than‐average MSE for both models, whereas the bottom example had higher‐than‐average MSE. At the plan level, DL‐MO yielded substantially better final plan quality than DL‐DO.

**FIGURE 5:**
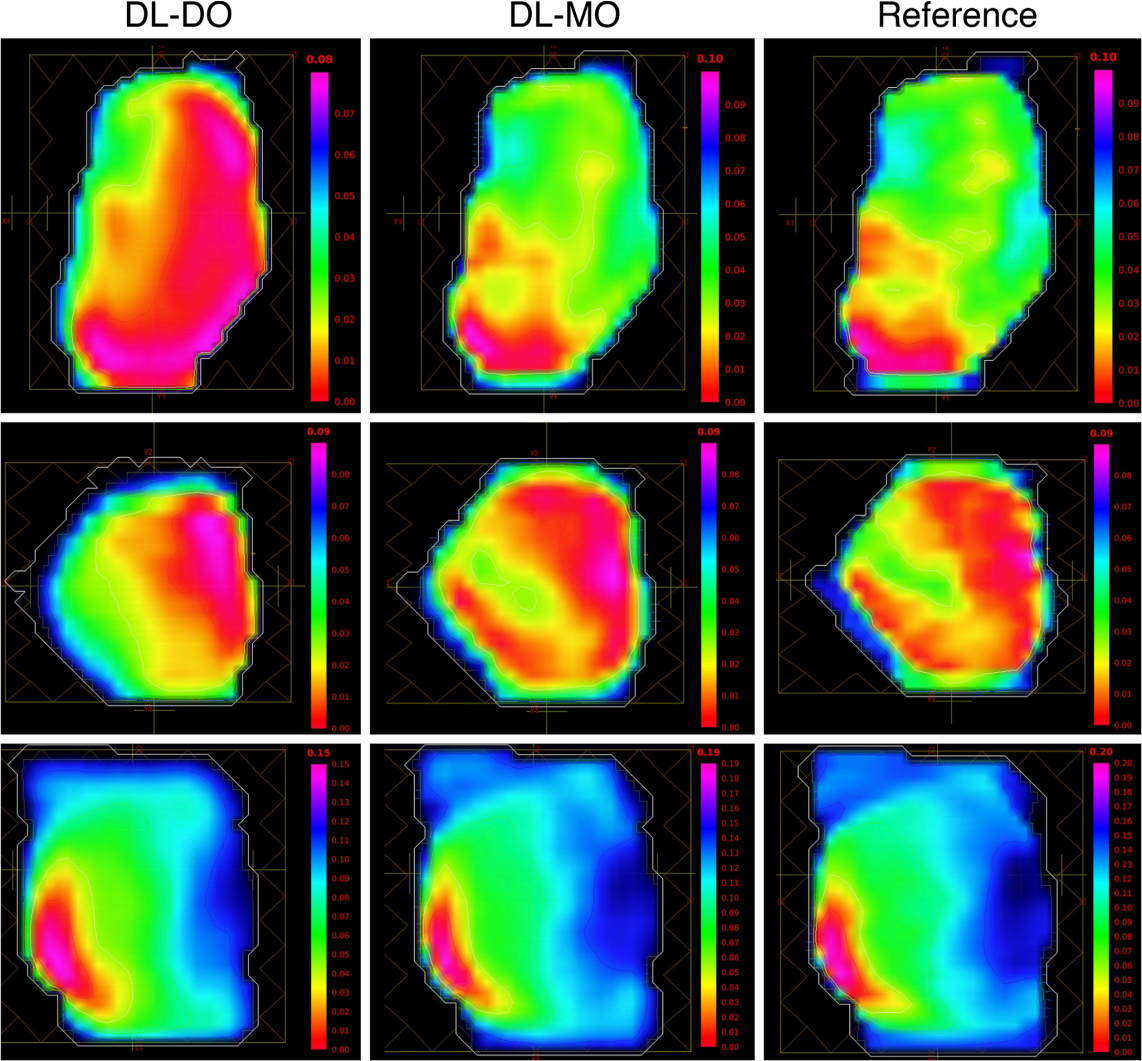
Visual assessment of the final predicted fluence maps used for plan generation in the TPS. For both DL‐DO and DL‐MO, the predicted fluence maps were obtained by applying M_1_ to the field doses predicted by the generator G. Columns show the fluence maps predicted by DL‐DO and proposed DL‐MO, together with the TPS reference fluence map; rows show three beams selected at random from the held‐out evaluation set. Fluence is shown in relative units. TPS, treatment planning system.

Quantitatively, DL‐MO improved both target coverage and hotspot control relative to DL‐DO and more closely approached the TPS reference (Table 1, Figure 6). In paired comparisons, DL‐MO significantly improved *D*_98_ and reduced *D*_2_ and *D*_max_ relative to DL‐DO, with median paired differences (DL‐MO—DL‐DO) of +12.31 pp, −3.43 pp, and −3.18 pp, respectively (Holm‐corrected paired Wilcoxon *p* < 0.0001 for all metrics). Relative to the TPS reference, DL‐MO showed only a small residual reduction in target coverage (median difference in *D*_98_: −1.66 pp; *p* = 0.014), although hotspot metrics remained elevated (*D*_2_ = +1.60 pp and *D*_max_ = +4.01 pp; both *p* < 0.001). In contrast, DL‐DO deviated substantially from the TPS reference across all evaluated metrics, with marked loss of coverage and higher hotspots (median differences: *D*_98_ = −13.62 pp, *D*_2_ = +4.90 pp, and *D*_max_ = +6.48 pp; all *p* < 0.001). This difference was also evident in representative dose distributions (Figure 7, Figure 8).

**TABLE 1:** PTV dose metrics for the final machine‐deliverable plans (n = 61) obtained by importing the predicted fluence maps of DL‐DO and DL‐MO into the TPS for leaf sequencing and AcurosXB dose recalculation, together with the TPS reference IMRT plans.

Metric (%)	DL‐DO	DL‐MO	TPS reference
*D*_98%_	78.59 ± 5.92	90.10 ± 3.74	91.62 ± 3.45
*D*_2%_	109.65 ± 2.40	106.28 ± 1.13	104.76 ± 1.00
*D*_max_	113.27 ± 3.03	110.32 ± 2.20	106.54 ± 1.45
*D*_mean_	100.00 ± 0.00	100.00 ± 0.00	100.00 ± 0.00

***Note***: Values are mean ± SD after renormalization to a mean target dose of 100%.

**Abbreviations**: PTV, planning target volume; TPS, treatment planning system.

**FIGURE 6:**
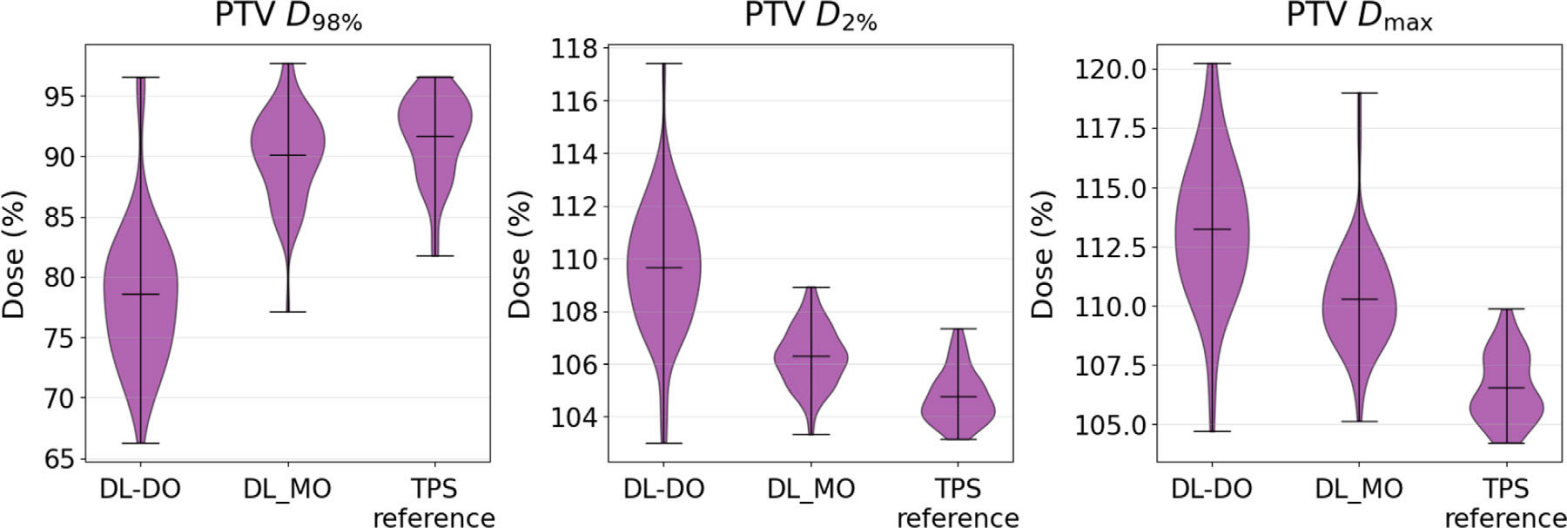
PTV dose metrics for the final machine‐deliverable plans (n = 61) obtained after import of fluence maps predicted by DL‐DO and DL‐MO into the TPS for leaf sequencing and AcurosXB dose recalculation, together with the TPS reference IMRT plans. Violin plots show the distributions after renormalization to a mean target dose of 100%. PTV, planning target volume.

**FIGURE 7:**
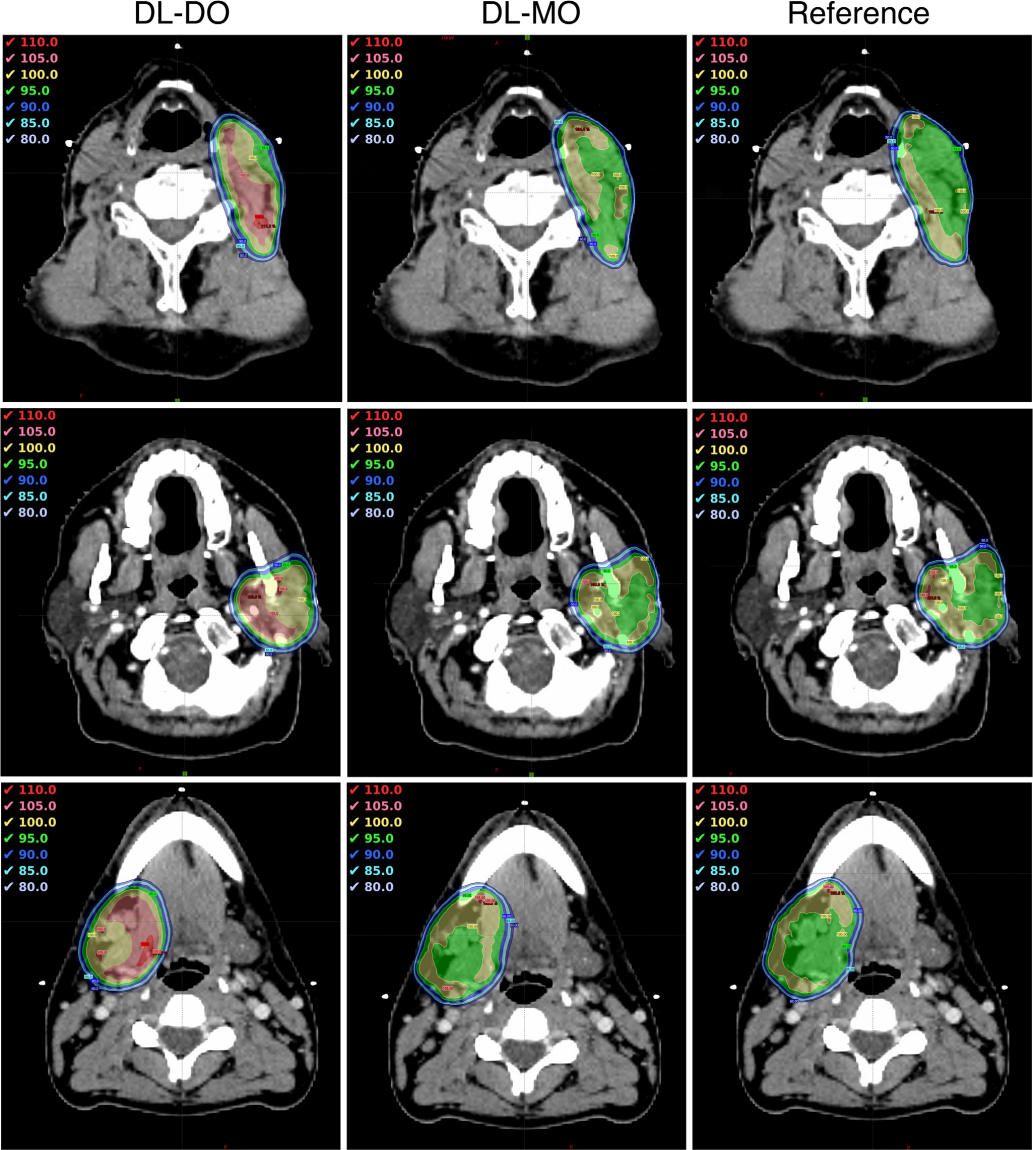
Axial dose slices for the final machine‐deliverable plans. Columns show the DL‐DO and DL‐MO plans obtained after import of the predicted fluence maps into the TPS for leaf sequencing and AcurosXB dose recalculation, together with the TPS reference IMRT plans; rows show randomly selected test patients. Dose was renormalized to a mean target dose of 100%, with isodose colorwash levels shown from 80% to 110%. TPS, treatment planning system.

**FIGURE 8:**
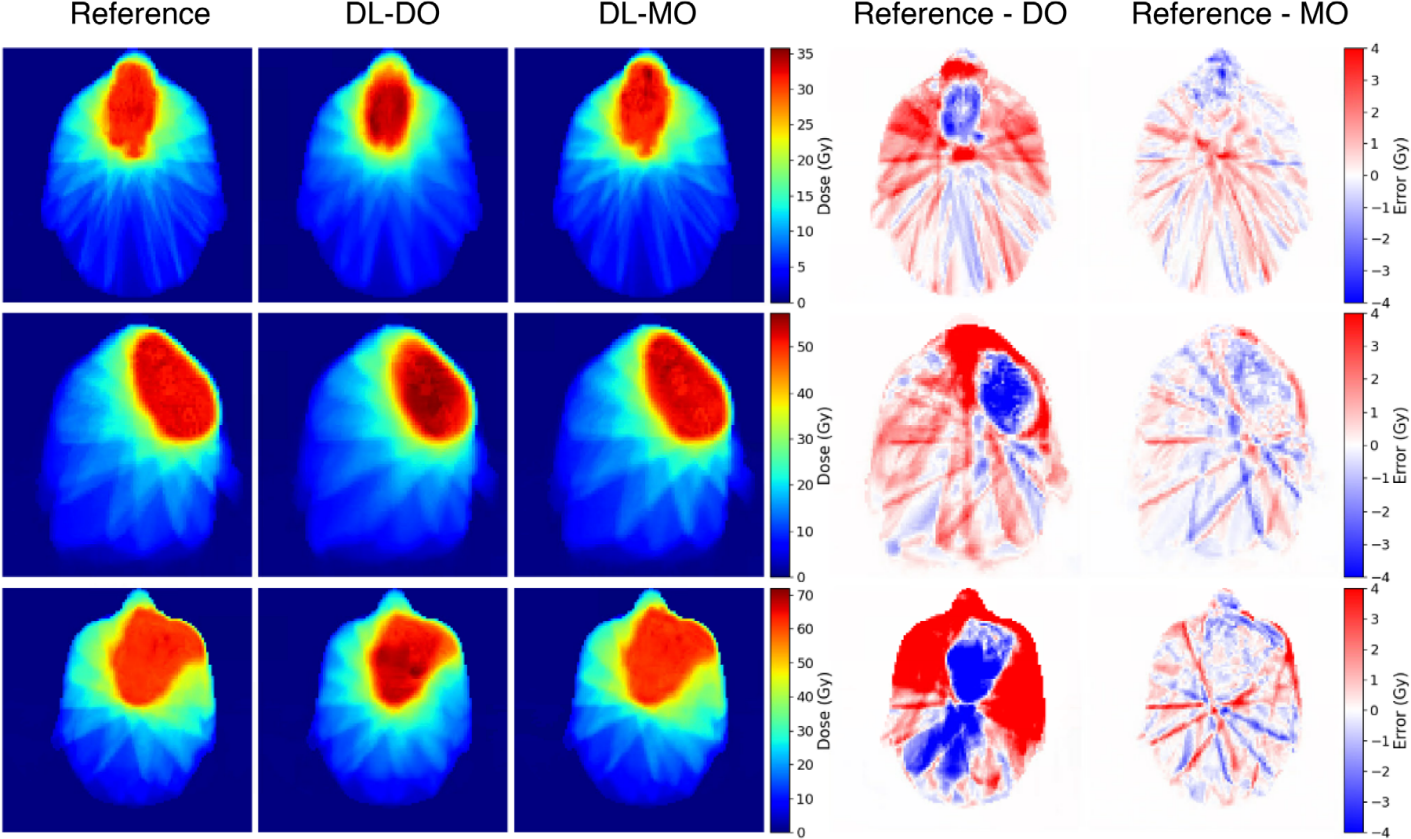
Final 3D dose distributions for the machine‐deliverable plans and voxel‐wise difference from the TPS reference, displayed as MIPs. Columns show the TPS reference IMRT plan, the DL‐DO and DL‐MO plans obtained after import of the predicted fluence maps into the TPS for leaf sequencing and AcurosXB dose recalculation, and the corresponding difference maps; rows show randomly selected test patients. Dose is shown in absolute units (Gy). TPS, treatment planning system; MIP, maximum‐intensity projection.

These quantitative differences were also reflected in an exploratory threshold analysis using evaluation thresholds of *D*_98_ > 90% and *D*_max_ ≤ 110%. The proportion of plans meeting both criteria was 72.1% for TPS reference IMRT, 4.9% for DL‐DO, and 31.1% for DL‐MO. For *D*_98_ > 90% alone, the corresponding rates were 72.1%, 4.9%, and 57.4%, whereas for *D*_max_ ≤ 110% they were 100%, 11.5%, and 45.9%, respectively.

Fluence‐map generation remained computationally efficient for both DL methods, with mean inference times below 1 s per fluence map.

Exploratory OAR dose metrics are summarized in Table 2. The numbers of eligible cases were 39 for the spinal cord, 10 for the brainstem, 23 for the parotid glands, and 13 for the submandibular glands. DL‐MO values were numerically close to the TPS reference values across all four evaluated OARs.

**TABLE 2:** Exploratory dosimetric evaluation of the most frequently delineated OARs in the final TPS‐recalculated plans.

OAR	Metric	*n*	DL‐DO	DL‐MO	TPS reference
Spinal cord	*D*_2%_ (Gy)	39	14.51 ± 8.69	14.99 ± 8.53	14.97 ± 8.55
Brainstem	*D*_2%_ (Gy)	10	17.67 ± 6.20	18.93 ± 6.27	18.60 ± 6.00
Parotid glands	*D*_mean_ (Gy)	23	5.16 ± 4.01	5.52 ± 3.70	5.48 ± 3.68
Submandibular glands	*D*_mean_ (Gy)	13	10.90 ± 5.63	11.87 ± 6.29	11.35 ± 6.09

***Note***: Values are mean ± SD and are reported as absolute dose in Gy. The number of eligible plans (n) varies by OAR because OAR availability differed according to anatomical treatment site.

**Abbreviations**: DL‐DO, deep‐learning dose‐only model; DL‐MO, deep‐learning multi‐objective model; OAR, organ at risk; SD, standard deviation; TPS, treatment planning system.

### 3.3 Ablation study

The ablation results are summarized in Table 3. Adding fluence supervision to the dose objective substantially improved final plan quality relative to dose‐only supervision, increasing mean *D*_98_ from 78.59% to 88.27% and reducing *D*_2_ and *D*_max_ from 109.65% and 113.27% to 106.95% and 111.27%, respectively. In contrast, combining dose supervision with the dose–fluence–dose consistency loss alone resulted in poorer performance than dose‐only supervision, with mean *D*_98_, *D*_2_, and *D*_max_ values of 73.48%, 111.65%, and 117.10%, respectively. The full DL‐MO objective achieved the best performance among the evaluated DL configurations, with further improvements over L *_dose_* + L *_FM_* for all three PTV metrics.

**TABLE 3:** Ablation study isolating the effects of the individual loss terms on final TPS‐recalculated plan quality (n = 61).

Metric (%)	DL‐DO (L *_dose_*)	L *_dose_* + L *_cyc_*	L *_dose_* + L *_FM_*	DL‐MO (all losses)
*D*_98_%	78.59 ± 5.92	73.48 ± 8.10	88.27 ± 4.35	90.10 ± 3.74
*D*_2_%	109.65 ± 2.40	111.65 ± 5.51	106.95 ± 1.83	106.28 ± 1.13
*D*_max_	113.27 ± 3.03	117.10 ± 4.93	111.27 ± 2.45	110.32 ± 2.20

***Note***: Values are mean ± SD after renormalization to a mean target dose of 100%.

**Abbreviations**: DL‐DO, dose‐only objective model; DL‐MO, multi‐objective model; PTV, planning target volume; TPS, treatment planning system.

### 3.4 Evaluation of *M_3_* on DL‐predicted fluence maps

The *M_3_* model and the clinical TPS dose engine AcurosXB both estimate dose from fluence maps. We therefore evaluated the agreement between *M_3_* and AcurosXB on the DL‐predicted fluence maps (Figure 2d).

Although *M_3_* achieved low error on TPS‐derived fluence maps in the stand‐alone evaluation, agreement with AcurosXB was lower on DL‐predicted fluence maps. The MAE between the dose distributions predicted by *M_3_* and calculated by AcurosXB was 0.435 ± 0.368. For DVH metrics, the largest discrepancy was observed for *D*_98_ (9.13 percentage points), whereas smaller differences were observed for *D*_2_ (1.19 percentage points) and *D*_max_ (0.52 percentage points).

## 4 DISCUSSION

This work investigated whether a novel cycle‐consistency training improves the deliverability of DL‐generated IMRT plans for lymphoma. In an end‐to‐end workflow, predicted fluence maps were imported into the TPS, leaf‐sequenced, and recalculated with AcurosXB to obtain the final machine‐deliverable plan. Compared with a baseline dose‐only model (DL‐DO), the proposed multi‐objective model (DL‐MO; dose supervision, fluence supervision and cycle consistency) significantly improved target coverage and reduced hotspots, although statistically significant differences from the TPS reference remained for all evaluated PTV metrics.

The numerically lower OAR doses observed for DL‐DO should not be interpreted as improved OAR sparing, because these values occurred alongside substantially poorer target coverage and greater target‐dose heterogeneity. In contrast, DL‐MO improved target coverage while maintaining OAR doses close to the TPS reference. The OAR analysis was exploratory, with organ‐specific sample sizes limited by variation in OAR availability across anatomical treatment sites.

Prior DL planning studies commonly report dose or fluence prediction accuracy, but these intermediate outputs do not necessarily translate to deliverable plans.^11^, ^12^, ^13^, ^14^, ^16^ A predicted 3D dose does not uniquely determine machine parameters, and fluence prediction alone does not guarantee a physically plausible modulation pattern once leaf sequencing and dose recomputation are applied. The presented results suggest that the proposed cycle‐consistency training helps close this gap by constraining optimization toward more deliverable solutions. In particular, DL‐MO produced fluence maps that more closely resembled the TPS reference and yielded higher‐quality machine‐deliverable plans (Figure 6, Figure 7), addressing a key limitation of prior work that reports only intermediate prediction accuracy.

The generator output is constrained to remain consistent after passing through a learned inverse mapping from field dose to fluence (*M_1_*) and a learned forward mapping from fluence to dose (*M_3_*). This constrains the generator to avoid unrealistic field‐dose patterns and promotes dose predictions that are more physically plausible and more readily representable by a fluence map. Consequently, the fluence maps imported into the TPS are better aligned with the predicted dose.

While *M_3_* and AcurosXB solve the same forward problem, agreement was lower on DL‐predicted fluence maps than on TPS‐derived inputs in the stand‐alone evaluation (Figure 2a,d). This is consistent with a distribution shift. Models *M_3_* and *M_1_* were trained in isolation on TPS‐optimized dose–fluence pairs, whereas during cycle‐consistency training they have to handle generator outputs that may fall outside this supervized training distribution. In particular, *M_3_* may encounter atypically modulated fluence patterns and *M_1_* locally unphysical field‐dose patterns. This likely contributes to the reduced agreement with AcurosXB and suggests that future work should improve the robustness of models *M_1_* and *M_3_*, for example by broadening the paired training data or by introducing additional constraints against non‐physical predictions. Structured residual patterns were visible in some stand‐alone *M_1_* predictions (Figure 3). Although the 2×2 transposed convolutions with stride 2 avoid unequal spatial coverage,^29^ checkerboard artefacts may still occur. Resize‐convolution, in which interpolation is followed by convolution, has been proposed as an alternative approach^29^ and should be investigated in future work.

The ablation analysis further clarifies how the individual loss terms contributed to final plan quality. Direct fluence supervision provided the principal stabilizing contribution within the multi‐objective framework. When the dose–fluence–dose consistency loss was applied without *ℒ_FM_*, the frozen *M_1_* and *M_3_* models processed generator predictions that could fall outside the distribution of TPS‐derived dose–fluence pairs used during their training. Under these conditions, consistency alone did not ensure agreement with the reference dose or fluence and resulted in poorer final plan quality than dose‐only supervision. In contrast, *ℒ_FM_* directly guided the generator towards field‐dose predictions that mapped to the reference fluence patterns. The additional improvement obtained with the full DL‐MO objective suggests that, once this mapping was stabilized by fluence supervision, the dose–fluence–dose consistency loss provided a complementary constraint favoring field‐dose predictions that were consistent with the learned dose–fluence mapping.

A further limitation is the quality and intent of the TPS reference plans. These IMRT plans were created in a standardized manner (fixed geometry and automated scripting) to provide a consistent reference and enable fair end‐to‐end comparison, but they were not clinically delivered plans and were not individually refined for maximal clinical quality. Consequently, the models learn the characteristics and quality of this specific standardized 15‐field IMRT replanning workflow, including any limitations introduced during replanning. The quality of these reference plans may therefore limit the absolute quality achieved by models trained on them, unless additional clinical objectives are incorporated. Generalizability to other beam arrangements, treatment techniques, institutions, or disease sites is expected, but would likely require additional validation and potentially retraining/finetuning. However, because DL‐DO and DL‐MO were trained and evaluated using the same reference plans, their comparison remains controlled and isolates the effect of the proposed training strategy. The limitation, therefore, does not affect the conclusions of the present study, including that the proposed cycle‐consistency approach improves the deliverability of predicted plans.

The learning objective includes partial plan replication (dose and fluence supervision). This does not prevent the generator from converging to alternative valid trade‐offs, as RT planning admits multiple Pareto‐optimal solutions for a given anatomy.^30^, ^31^, ^32^ We therefore weighted the cycle term to emphasize physical consistency and deliverability while retaining dose supervision to prevent drift toward implausible solutions. This perspective is clinically relevant because deliverability constraints narrow the solution space to realizable plans without enforcing a single unique solution. Consistent with this, Figure 8 (last column) shows minimal differences within the target, whereas larger differences are observed along individual beam paths, suggesting that the model converges to a solution distinct from the reference plan while more closely matching the reference target‐dose distribution than DL‐DO.

From a translational standpoint, the framework provides fast, automated generation of machine‐deliverable IMRT plans for a challenging, heterogeneous lymphoma population in which targets vary widely in location, extent, and fractionation. The ability to produce multiple machine‐deliverable candidate plans without manual intervention may support use cases such as rapid baseline plan generation, plan‐quality triage, and producing multiple alternatives for clinician review. Because deliverability is enforced during learning, the framework could be extended to incorporate additional planning criteria (e.g., OAR constraints, protocol‐specific DVH goals, or patient‐specific risks^33^, ^34^, ^35^) as extra inputs while preserving a feasible solution space.

## 5 CONCLUSION

We developed a novel cycle‐consistency training approach for automated IMRT planning in a heterogeneous lymphoma cohort. When evaluated as machine‐deliverable plans within the TPS, the proposed method produced significantly improved DVH metrics and dose error (MAE) relative to conventional dose‐only supervision. DL‐MO yielded plan quality closer to the TPS reference than DL‐DO, although statistically significant differences remained for all evaluated PTV metrics. This deliverability‐aware framework could be extended to incorporate additional constraints (e.g., protocol‐specific DVH goals or patient‐specific risk objectives) while enabling fast, automated generation of clinically relevant, machine‐deliverable plans in the TPS.

## AUTHOR CONTRIBUTIONS

**Denis Kutnár**: Conceptualization; methodology; software; validation; formal analysis; investigation; data curation; visualization; writing—original draft; writing—review and editing; project administration. **Ivan Richter Vogelius**: Supervision; conceptualization; methodology; validation; clinical evaluation; writing—review and editing. **Hristo Atanasov Georgiev**: Investigation; conceptualization; validation; software; writing—review and editing. **Lena Specht**: Resources; supervision; validation; writing—review and editing. **Jens Petersen**: Supervision; conceptualization; methodology; software; validation; writing—review and editing; project administration.

## CONFLICT OF INTEREST STATEMENT

The authors declare no conflicts of interest.

## ETHICS STATEMENT

This study was approved by the Ethics Committee of the Capital Region, Copenhagen, Denmark, approval R‐23054402.

## Data Availability

The data that support the findings of this study are not publicly available due to patient privacy and ethical restrictions. Data may be made available from the corresponding author upon reasonable request and subject to institutional approval.
